# Potentiation of Sodium Metabisulfite Toxicity by Propylene Glycol in Both *in Vitro* and *in Vivo* Systems

**DOI:** 10.3389/fphar.2018.00161

**Published:** 2018-02-28

**Authors:** Jean Yoo, Yeon-Mi Lim, Haewon Kim, Eun-Ji Kim, Doo-Hee Lee, Byeongwoo Lee, Pilje Kim, Seung Do Yu, Hyun-Mi Kim, Byung-Il Yoon, Ilseob Shim

**Affiliations:** ^1^Environmental Health Research Department, National Institute of Environmental Research, Incheon, South Korea; ^2^Environmental Measurement and Analysis Center, National Institute of Environmental Research, Incheon, South Korea; ^3^College of Veterinary Medicine and Institute of Veterinary Science, Kangwon National University, Chuncheon, South Korea

**Keywords:** potentiation, sodium metabisulfite, propylene glycol, cytotoxicity, inhalation

## Abstract

Many consumer products used in our daily lives result in inhalation exposure to a variety of chemicals, although the toxicities of the active ingredients are not well known; furthermore, simultaneous exposure to chemical mixtures occurs. Sodium metabisulfite (SM) and propylene glycol (PG) are used in a variety of products. Both the cytotoxicity and the sub-acute inhalation toxicity of each chemical and their mixtures were evaluated. Assays for cell viability, membrane damage, and lysosome damage demonstrated that SM over 100 μg/ml induced significant cytotoxicity; moreover, when PG, which was not cytotoxic, was mixed with SM, the cytotoxicity of the mixture was enhanced. Solutions of 1, 5, and 20% SM, each with 1% PG solution, were prepared, and the whole body of rats was exposed to aerosols of the mixture for 6 h/day, 5 days/week for 2 weeks. The rats were sacrificed 1 (exposure group) or 7 days (recovery group) after termination of the exposure. The actual concentration of SM in the low-, medium-, and high-exposure groups was 3.91 ± 1.26, 35.73 ± 6.01, and 80.98 ± 5.47 mg/m^3^, respectively, and the actual concentration of PG in each group was 6.47 ± 1.25, 8.68 ± 0.6, and 8.84 ± 1.77 mg/m^3^. The repeated exposure to SM and PG caused specific clinical signs including nasal sound, sneeze, and eye irritation which were not found in SM single exposure. In addition, the body weight of treatment group rats decreased compared to that of the control group rats in a time-dependent manner. The total protein concentration and lactate dehydrogenase activity in the bronchoalveolar lavage fluid (BALF) increased. Histopathological analysis of the lungs, liver, and nasal cavity was performed. Adverse effects were observed in the nasal cavity, with squamous cell metaplasia identified in the front of the nasal cavity in all high-exposure groups, which completely recovered 7 days after exposure was terminated. Whereas inhalation of SM for 2 weeks only reduced body weight in the high-dose group, inhalation of SM and PG mixtures for 2 weeks significantly decreased body weight and induced metaplasia of the respiratory epithelium into squamous cells in the medium- and high-dose groups. In conclusion, PG potentiated the toxicity of SM in human lung epithelial cells and the inhalation toxicity in rats.

## Introduction

Many types of chemicals are used in household products, including deodorants, air fresheners, cosmetics, disinfectants, paints, and hair spray. During our daily activities, spray-type household products are recognized as the major source of inhalation exposure to chemicals (Hahn et al., 2010; Shim et al., 2013), although they are not well studied despite their wide usage. Sodium metabisulfite (Na_2_S_2_O_5_; SM), also known as disodium salt, sodium pyrosulfite, disodium sulfite, sodium sulfite anhydrous, or sodium disulfite, is an inorganic sulfite used widely as a preservative to combat the proliferation of microorganisms; additionally, it has antioxidant properties in some wines and foods (Jamieson et al., 1985; Ercan et al., 2015) and is used as a disinfectant or an antioxidant in cosmetic products and some pharmaceuticals (Noorafshan et al., 2013). SM is a white crystalline or powder solid chemical with a slight sulfur odor and this chemical becomes a corrosive acid when they are mixed with water. As they become a corrosive acid, SM can cause cough and wheezing when inhaled (Steiner et al., 2008). In previous studies, SM was reported to trigger asthma through the induction of bronchoconstriction (Stevenson and Simon, 1981; Nichol et al., 1989; Nannini and Hofer, 1997). The previous report indicated that the response to SM occurs as a result of the effects of sulfur dioxide, which affects the sensory nerves and causes mediator release (Wright et al., 1990).

Propylene glycol (PG), a colorless viscous liquid, is also odorless and soluble in water (Kulo et al., 2012). This chemical is hygroscopic and miscible with water, acetone, and chloroform; therefore, it is widely used as the solvent and diluent in many products mixed with various chemicals (Cruz et al., 2002). Emulsifiers are a common component of many consumer products, and PG is a representative emulsifier, which is used widely in our daily life. Based on the information given by the Household Produces Database^1^, PG is used in creamy soaps, toothpastes, air fresheners, cleaners, stain removers, and in foods as a preservative. Previous studies reported that PG was relatively safe and non-toxic when used as a single chemical (Gaunt et al., 1972; Ruddick, 1972; Wilson et al., 2005).

Although exposure to chemical mixtures commonly occurs and is considered to be important in toxicology, the toxicities caused by inhalation are less well studied (Cassee et al., 1996; Feron et al., 2002; Yang et al., 2004). In particular, compared with each single chemical, *in vivo* studies of the toxicities of mixtures have not been carried out sufficiently to obtain full comprehension of the health effects (Kortenkamp, 2008; Altenburger et al., 2013). Several commercially available products that contain SM are also known to include PG as a preservative. It is therefore of concern that some of these products may be dispersed in the air in an aerosol form, and toxic aerosols could cause serious diseases to the lungs, such as interstitial lung disease (Kim et al., 2014, 2016; Park et al., 2014; Paek et al., 2015).

Toxicological interactions such as addition, synergy, potentiation, and antagonism could occur when two chemicals are exposed to organisms simultaneously (Bae et al., 2001; Grenier and Oswald, 2011; Jarvis et al., 2014). Therefore, evaluation of toxicity of chemical mixtures could not be derived by simple addition of their individual toxicity (Rizzati et al., 2016). For example, zinc oxide nanoparticles at non-toxic concentration potentiates the cytotoxicity of copper nanoparticles (Li et al., 2015). In addition, many researchers have revealed the potentiation of toxic effects of some chemicals in both *in vitro* and *in vivo* systems, recently (Pérez Martín et al., 2014; Tabet et al., 2016; Yang et al., 2016).

However, to the best of our knowledge, no studies have been conducted to observe the *in vitro* and *in vivo* reactions of SM or to study the inhalation toxicity of the chemical mixture of SM with PG. Therefore, this study aimed to evaluate the toxicological effects of SM and PG in human alveolar basal epithelial cells (A549), and to evaluate whole-body inhalation toxicity caused by single exposure of SM and by the mixture of SM with PG. We also compared the differences in toxicity between SM and the mixture of SM and PG both *in vitro* and *in vivo*.

## Materials and Methods

### Chemicals

Sodium metabisulfite and PG used in the present study were purchased from Sigma–Aldrich Co., Ltd. (St. Louis, MO, United States).

### *In Vitro* Study With A549 Cells

For the *in vitro* assay, A549 human alveolar epithelial cells were purchased from American Type Culture Collection (ATCC). The cells were cultured in RPMI 1640 (Thermo Fisher Scientific Inc., Waltham, MA, United States) culture medium supplemented with 10% v/v fetal bovine serum and 1% penicillin–streptomycin at 37°C, 5% CO_2_/95% air.

To evaluate the toxicity of SM and PG to A549 cells, the cells were plated in 96-well plates (1 × 10^5^ cells/well) and incubated for 24 h. The single chemical toxicity test for SM and PG was performed at different concentrations (50–500 μg/ml for SM and 31.25–1000 μg/ml for PG) after incubation for another 24 h. The toxicity of the SM+PG mixture was also evaluated by the incubation of 200 μg/ml SM and 31.25–1000 μg/ml PG for 24 h. Cell morphology was observed by optical microscopy, and cell viability was evaluated by EZ-Cytox staining (DoGenBio Co., Ltd.) for the measurement of 3-(4,5-dimethylthiazol-2-yl)-2,5-diphenyltetrazolium bromide (MTT). The membrane integrity was checked by using EZ-lactate dehydrogenase (LDH) (DoGenBio Co., Ltd.) to measure the LDH level. Neutral red assay was done by measuring the accumulation of the neutral red dye in uninjured cell lysosomes, and a neutral red assay kit Tox4 (Sigma–Aldrich Co., Ltd., St. Louis, MO, United States) was used to evaluate the normal lysosome amount.

The clonogenic assay was conducted to observe cell growth, to identifying the colony forming ability, and to evaluate the cell division ability. Therefore, the cells were plated in 6-well plates (200 cells/well, in a total media volume of 4 ml) and incubated for 24 h. The cells were then treated with SM (200 μg/ml) and PG (31.25–1000 μg/ml). After 7 days of incubation, the medium was removed, the plates were then gently washed with PBS, fixed with methanol for 10 min on ice, and stained with 0.5% crystal violet.

### *In Vivo* Inhalation Study

#### Animals

Six-week-old-specific-pathogen-free (SPF) male Sprague-Dawley rats were purchased from Orient Bio Inc. (Seongnam, South Korea), and maintained in our laboratory animal facility at 21 ± 3°C, 50 ± 20% relative humidity, and a 12 h light/dark cycle. The animals were acclimatized for at least 1 week prior to the beginning of treatment. The animals were divided into four and five groups in the SM and SM+PG sub-acute exposure studies, respectively. The body weights were measured twice per week from the beginning of the study until the animals were sacrificed, and general symptoms and signs were monitored during exposure period. All animal experiments were accomplished in accordance with the guidelines of the Institutional Animal Care and Use Committee in National Institute of Environmental Research (South Korea).

#### Inhalation Exposure

After a 1 week acclimatization period, the rats were subjected to the inhalation exposure assay. Temperature was controlled during the exposure period (temperature, 21 ± 3°C; humidity, 50 ± 20%, and 12 h light/dark cycle). For the SM-treated sub-acute inhalation exposure study, the rats were exposed to 5 mg/m^3^ (low), 20 mg/m^3^ (medium), 100 mg/m^3^ (high) SM for 6 h/day, 5 days/week, for 14 days. For the study of the SM+PG mixture inhalation, the rats were divided into five groups (16 rats/group): the control, vehicle control (1% PG aqueous solution), low (1% PG aqueous solution + 1% SM aqueous solution), medium (1% PG + 5% SM), and high (1% PG + 20% SM) groups. We prepared an inhalable aerosol of those solutions by using an atomizer with clean air flow of 250 l/min. The rats were exposed for 6 h/day, 5 days/week, for 2 weeks (14 days). In each group, half of the rats were euthanized 1 day after the final exposure, with the remaining half sacrificed 7 days after the final exposure to monitor the recovery.

### SM and PG Monitoring

All the inhalation parameters were monitored during exposure, including temperature, humidity, air flow, and air pressure, by Model VT3-X15, Sibata Scientific Technology Ltd. (Saitama, Japan). The concentration of SM was monitored by sampling the chamber air using a SIP-32L (SIBATA) and comparing the weight difference of the membrane (T60A20, Φ55). The particle size was measured by using a portable aerosol spectrometer (Model 1.109, Grimm Aerosol Technik, GmbH & Co. KG, Ainring, Germany). The exposure to PG was measured in accordance with the National Institute for Occupational Safety and Health (NIOSH) analysis manual 2355, by sample collection with an XAD-7 OVS tube and analysis with gas chromatography-flame ionization detector (GC–FID). The GC/FID system was Agilent 6890 (Agilent Technologies Inc., Santa Clara, CA, United States) and the GC column used for sample analysis was a DB-WAX (30 m × 0.25 mm × 0.25 μm) from J&W Scientific Inc. (Santa Clara, CA, United States).

### Bronchoalveolar Lavage (BAL) Analysis

To evaluate the toxicity caused by inhalation in the lungs, all rats were euthanized and the BAL fluid (BALF) was analyzed. Lavage fluid from the rat lungs was obtained three times with 4 ml calcium- and magnesium-free phosphate-buffered saline (PBS, pH 7.4). BALF was centrifuged at 1500 rpm for 10 min in a Hanil Union 32R (Incheon, Korea) and the supernatants were collected and stored at -80°C until use. The bicinchoninic acid (BCA) protein assay (Intron, Korea) and LDH assay were conducted by using a Cytotoxicity Assay Kit (Daeil Lab Service Co., Ltd., Korea). Analyses of the expression of tumor necrosis factor-alpha (TNF-α), interleukin-6 (IL-6), transforming growth factor-beta1 (TGF-β1), interleukin-1beta (IL-1β), and monocyte chemoattractant protein-1 (MCP-1) were conducted by using ELISA kits provided by R&D systems (Minneapolis, MN, United States) to evaluate the differences in inflammatory cytokine levels caused by inhalation. After centrifugation, the BALF pellets were collected and the number of cells in the BALF samples was counted by using a Vi-Cell^®^ XR analyzer (Beckman Coulter, Brea, CA, United States). Based on the cell number results, 50 μl of the ready-made samples was diluted to 2 × 10^5^ cells/ml and injected to the slide-set in a Shandon Cytospin (Shandon, Pittsburgh, PA, United States) and the slides were stained with Diff-Quik.

### Histopathological Analysis

After inhalation of SM and the mixture of SM+PG for 2 weeks, the animals were sacrificed under anesthesia. After gross examination, liver, lung, and nasal cavity were fixed in 10% neutral-buffered formalin. Nasal cavity was decalcified with a decalcification solution and then cross-sectioned at three section levels of nasal cavity: section level I (immediately posterior to the upper incisor teeth), section level II (between the incisive papillae and the first palatal ridge), and section level III (middle of the molar teeth). After the routine tissue processing, the tissues were embedded in paraffin, and then sectioned in 3 μm thickness. The tissue sections were then stained with hematoxylin and eosin (H&E) and histological examination was performed under a light microscope (Olympus BX41, Tokyo, Japan). Lesions were graded separately depending on the severity of the lesions by pathologists.

### Statistical Analysis

Statistical analyses were computed by using PASW Statistics 18 (SPSS Inc., Korea). Nonparametric statistical tests were used to compare the SM- and PG-treated groups compared with the control groups, and the values are expressed as the mean ± standard deviation (SD). One-way multiple variance of analysis (ANOVA) test followed by Student’s *t*-test was used to compare the exposure groups with control group and to observe whether the *P*-value was <0.05, 0.01, or 0.001.

## Results

### *In Vitro* Assay

**FIGURE 1:**
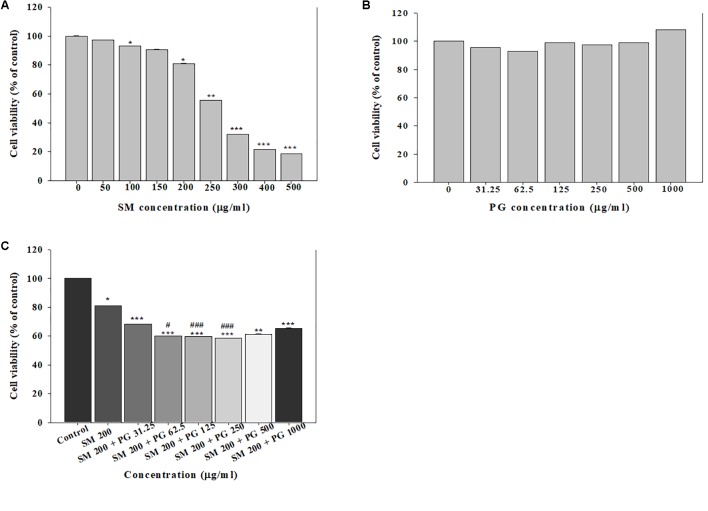
The cell viability was measured by the MTT assay. The human lung epithelial cells (A549) were exposed to different concentrations of **(A)** sodium metabisulfite (SM), **(B)** propylene glycol (PG), and **(C)** a mixture of SM and PG for 24 h. The vales are reported as the mean ± SE (Student’s *t*-test, ^∗^*P* < 0.05, ^∗∗^*P* < 0.01, and ^∗∗∗^*P* < 0.00 vs. control).

To assess the toxicity in A549 cells, the cells were treated with SM alone or with PG, and the MTT, LDH, and neutral red assays were performed. The treatment with SM alone to A549 cells induced a dose-dependent decrease in cell viability (by the MTT assay) at the concentrations above 100 μg/ml. The IC_50_ value was determined as 281.5 μg/ml (**Figure 1A**), although no differences in the cell membranes and lysosomes were found between the groups. The single administration of PG caused no effects at all tested concentrations (31.25–1000 μg/ml), which suggested that no cytotoxicological effects were caused in A549 cells in the concentration range (**Figure 1B**). Exposure to the SM+PG mixture was carried out with 200 μg/ml SM (the cell viability was about 80% of the control group) and multiple PG dose mixtures. Cell viability decreased in all groups treated with the SM+PG mixture compared to that in the control group (**Figure 1C**), although no differences were observed in the cell membranes and lysosomes.

**FIGURE 2:**
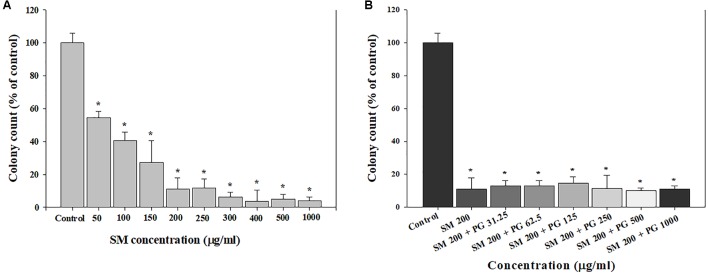
The inhibition of cell colony formation induced by the SM in human lung epithelial cells. **(A)** SM and **(B)** SM+PG mixture. A549 cells were exposed to chemicals for 7 days. The values are reported as the mean ± SE (Student’s *t*-test, ^∗^*P* < 0.05 vs. control).

Clonogenic assays were used to evaluate the chronic effect of treatment with SM and SM+PG on A549 cells. First, 200 cells/well were seeded; after 24 h, the cells were treated with 50–1000 μg/ml SM or a mixture of 200 μg/ml SM and 31.25–1000 μg/ml PG, and then incubated for 7 days. After 7 days, the total number of colonies decreased markedly in a dose-dependent manner by SM treatment (**Figure 2A**), although different doses of PG with 200 μg/ml SM did not inhibit colony formation (**Figure 2B**).

### SM Sub-acute Inhalation Study

To investigate the toxicity of the sub-acute exposure of SM, 14-day inhalation studies were conducted. The rats were divided into four groups and allowed to inhale fresh air (negative control), 5, 20, and 100 mg/m^3^ of SM for 6 h, for 5 days/week for 2 weeks (a total of 10 days): control, low, medium, and high groups, respectively. The actual exposure concentration of SM was determined as 5.5 ± 2.4, 29.3 ± 7.7, and 110 ± 38.9 mg/m^3^ in the low-, medium-, and high-exposure groups, respectively. The particle size distribution of SM was measured by the multi-stage impactor and the mass median average diameter (MMAD) was in the range of 1.95–2.32 μm, with a geometric standard deviation (GSD) of 1.71–1.92 in each chamber, which matched the condition of the OECD Guideline for Testing of Chemicals.

**FIGURE 3:**
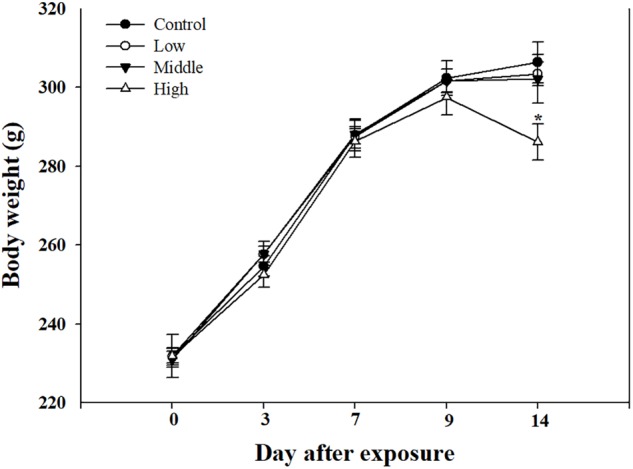
The body weight changes in rats exposed to sub-acute SM. Male rats inhaled aerosolic SM for 2 weeks. The values are reported as the mean ± SE (Student’s *t*-test, ^∗^*P* < 0.05 vs. control).

**FIGURE 4:**
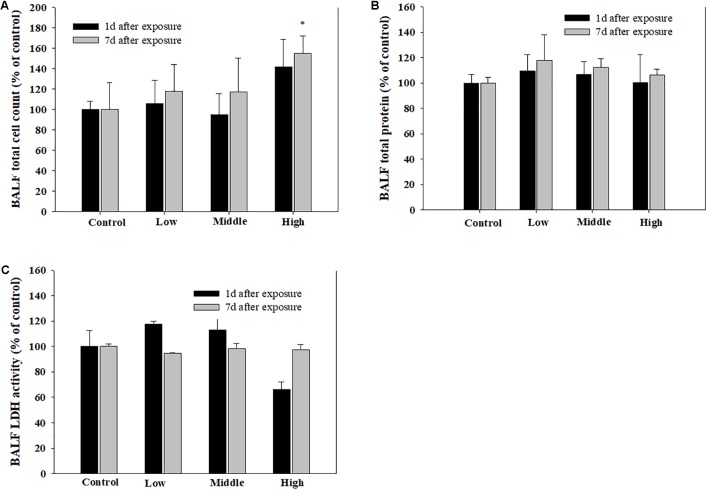
The pulmonary toxicity induced by sub-acute inhalation of the SM in rats. **(A)** Total cell count in bronchoalveolar lavage fluid (BALF), **(B)** total protein (TP) content in BALF, and **(C)** lactate dehydrogenase (LDH) activity in BALF. Male rats inhaled aerosolic SM for 2 weeks. The values are reported as the mean ± SE.

**FIGURE 5:**
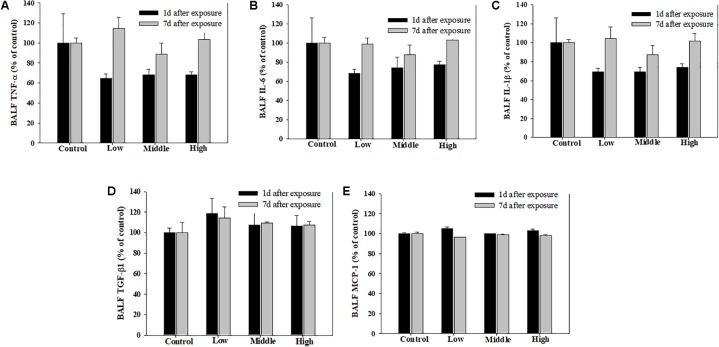
Pulmonary inflammation induced by repeated inhalation of the SM in rats. **(A)** Tumor necrosis factor-alpha (TNF-α), **(B)** interleukin-6 (IL-6), **(C)** interleukin-1beta (IL-1β), **(D)** transforming growth factor-beta1 (TGF-β1), **(E)** monocyte chemoattractant protein-1 (MCP-1) in BALF. Male rats inhaled aerosolic SM for 2 weeks. The values are reported as the mean ± SE.

Compared with the rats in the low- and medium-exposure groups (**Figure 3**), the body weight significantly decreased in the high-exposure group on the 14th day of exposure, although no other clinical symptoms were observed during the exposure. In the examination of the BAL fluid samples of the rats in each group, the total number of cells increased in the high-recovery group (measured 7 days after the final exposure) (**Figure 4A**), but no clear difference was found for the TP and LDH assays in all exposure groups (measured 1 day after the end of exposure) and the recovery groups (**Figures 4B,C**). The sub-acute exposure treatment groups resulted in no clear changes in all the lung inflammatory responses factors (**Figure 5**).

**FIGURE 6:**
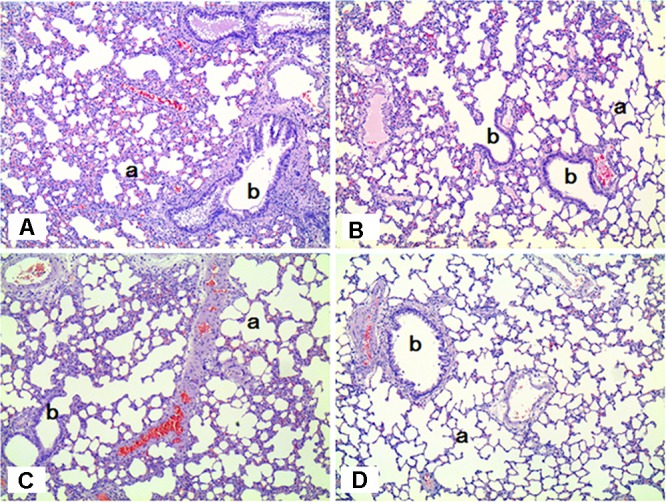
Histopathological staining of the lungs in male rats exposed to SM for 2 weeks by inhalation. No specific abnormal findings were noted in the lungs. a, alveoli; b, bronchiole. **(A)** Control group; **(B)** low-exposure group; **(C)** medium-exposure group; **(D)** high-exposure group; H&E staining. Magnification = ×100 for all images.

The histopathological results in the sub-acute toxicity study were shown in **Table 1**. In the liver, multifocal infiltration of mononuclear cells was observed in the control and SM treatment groups without notable difference in the incidence rate and severity among the groups. Focal bile ductile hyperplasia was noted in one rat out of the four in the low-dose group. Diffuse type of steatosis was evident in a rat of the medium- and high-dose groups, but it was minimal in severity. In the lungs, no specific abnormal findings were observed, although focal mineralization in the arterial wall was noted in a rat of the high-dose group (**Figure 6**).

**Table 1:** The summary of the histopathological results of rats repeatedly inhaled with sodium metabisulfite (SM).

Organ/histopathology	Group	Control	Low	Middle	High
	Dose (mg/m^3^)	0	5	20	100
	**No. examined**	**4**	**4**	**4**	**4**

**Liver**					
No specific lesion		1 (25.0)	1 (25.0)	1 (25.0)	0 (0.00)
Cell infiltration, mononuclear or mixed		2 (50.0)	3 (75.0)	2 (50.0)	3 (75.0)
*Grades: minimal*		2	3	2	3
Cell infiltration, lymphocytic, portal		1 (25.0)	1 (25.0)	0 (0.00)	1 (25.0)
*Grades: minimal*		1	1	0	0
*mild*		0	0	0	1
Bile duct hyperplasia, focal		0 (0.00)	1 (25.0)	0 (0.00)	0 (0.00)
*Grades: minimal*		0	1	0	0
Steatosis, diffuse		0 (0.00)	0 (0.00)	1 (25.0)	1 (25.0)
*Grades: minimal*		0	0	1	1

**Lung**					
No specific lesion		4 (100)	4 (100)	4 (100)	3 (75.0)
Arterial wall mineralization, focal		0 (0.00)	0 (0.00)	0 (0.00)	1 (25.0)
*Grades: minimal*		0	0	0	1

### Mixture Inhalation Study

As PG is known to be widely used as an emulsifier, we tried to evaluate the toxicological effect of the mixture of SM+PG. For this, rats were divided into five groups: control, vehicle control, low-, medium-, and high-exposure groups. The actual concentration of SM in the chamber was measured as 3.91 ± 1.26, 35.73 ± 6.01, and 80.98 ± 5.47 mg/m^3^ in the low-, medium-, and high-exposure groups, respectively, and the actual concentration of PG was measured as 5.5 ± 1.07 mg/m^3^ (vehicle control), 6.47 ± 1.25 mg/m^3^ (low), 8.68 ± 0.6 mg/m^3^ (medium), and 8.84 ± 1.77 mg/m^3^ (high) in each of the stated groups. The MMADs were between 1.70 and 2.75 μm with a GSD from 1.69 to 2.08 in each chamber.

**FIGURE 7:**
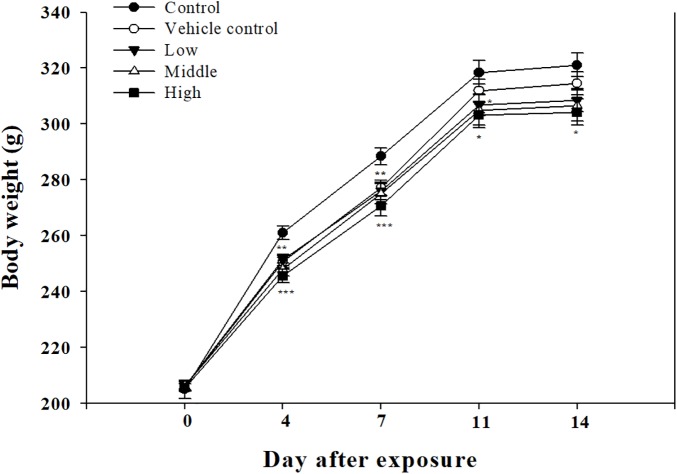
Body weight changes in rats exposed to SM and PG mixture. The male rats inhaled aerosolic SM and PG for 2 weeks. The analysis of aerosolic particle size distribution. Mean ± SE (Student’s *t*-test, ^∗^*P* < 0.05, ^∗∗^*P* < 0.01, and ^∗∗∗^*P* < 0.001 vs. control).

Clinical symptoms such as nasal sounds, sneezes, and eye irritations which were not observed in the SM single exposure study were observed in the rats exposed to the high SM+PG mixture. The body weight of the high-dose group rats was significantly decreased compared to that of the control group (**Figure 7**). The analyses of the blood and serum from the rats indicated that all results were within the normal ranges with no significant differences between the control and the exposure groups (data not shown).

**FIGURE 8:**
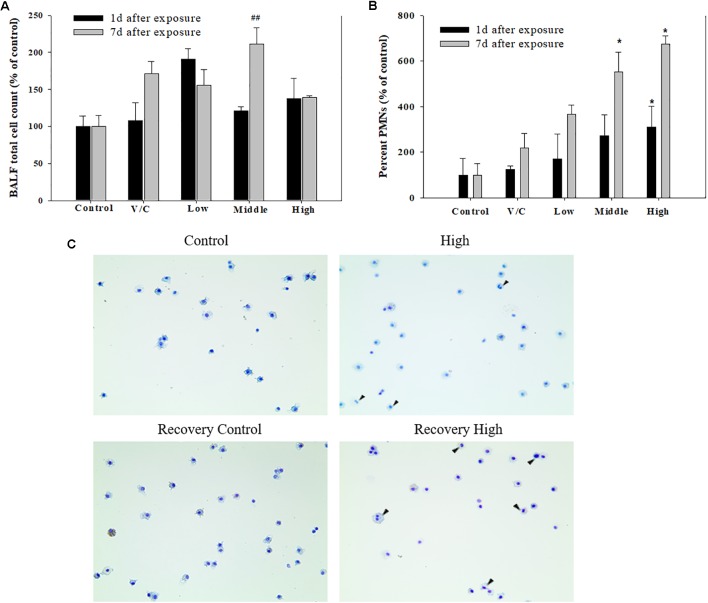
Pulmonary inflammation induced by repeated inhalation for 2 weeks of a mixture of SM and PG in male rats. **(A)** Total cell count in BALF. **(B)** Polymorphonuclear leukocyte (PMN) count in BALF. **(C)** Diff-Quick staining of BALF cells. Arrowhead indicates PMNs. The values are presented as the mean ± SE (one-way ANOVA followed by Tukey’s multiple comparison test, ^#^*P* < 0.05, ^##^*P* < 0.01, and ^###^*P* < 0.001, vs. control).

**FIGURE 9:**
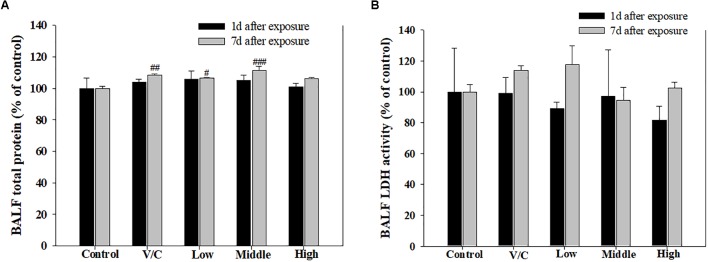
Pulmonary toxicity induced by repeated inhalation of a mixture of SM and PG for 2 weeks in male rats. **(A)** TP content in BALF and **(B)** level of LDH in BALF. The values are presented as the mean ± SE (one-way ANOVA followed by Tukey’s multiple comparison test, ^#^*P* < 0.05, ^##^*P* < 0.01, and ^###^*P* < 0.001 vs. control).

**FIGURE 10:**
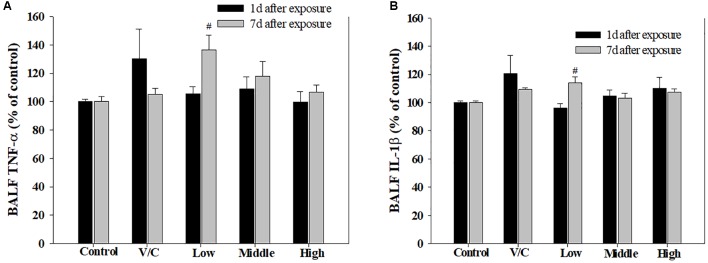
Changes in inflammatory cytokines induced by repeated inhalation of a mixture of SM and PG for 2 weeks in male rats. **(A)** Tumor necrosis factor-alpha (TNF-α), **(B)** interleukin-1beta (IL-1β) in BALF. The values are presented as the mean ± SE (one-way ANOVA followed by Tukey’s multiple comparison test, ^#^*P* < 0.05 vs. control).

To observe the toxicological effects on the rat lung caused by inhalation of SM+PG mixtures, the total numbers of BAL fluid and polymorphonuclear neutrophil (PMN) cells were counted. The total number of cells in the medium-recovery group was increased significantly and there was an increase in the low-recovery group without significant difference (**Figure 8A**). The number of PMNs was significantly increased in the medium and high groups (**Figures 8B,C**). In addition, the total protein (TP) and LDH levels of BAL fluid samples were measured; slight increases in the low- and medium-recovery groups were found (**Figure 9**). Various inflammation-related cytokines were measured and IL-1β and TNF-α were found to be increased in the low-dose recovery group, which suggested that SM may have caused prolonged inflammation in the rat lungs (**Figure 10**).

**FIGURE 11:**
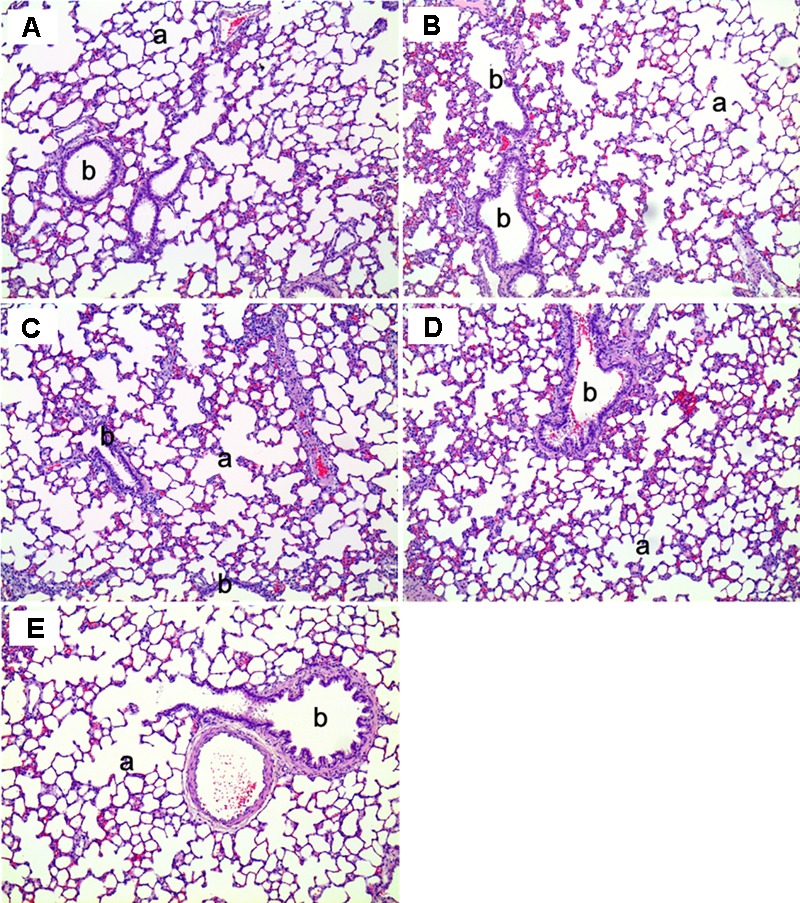
The histopathology of the lungs in male rats exposed to the mixture of SM and PG by aerosol inhalation for 2 weeks. The results of **(A–E)** are the results for the control, vehicle control, low, medium, and high groups, respectively. No pathological findings resulted from the exposure of the test materials. a, alveoli; b, bronchioles. H&E staining. Magnification = ×200 for all images.

Histopathological examinations of lung, liver, and nasal cavity were performed in the rats exposed to the mixture of SM and PG for the sub-acute toxicity study (**Table 2**). In the liver, multifocal mononuclear cell infiltration was observed in all treatment groups as well as in the non-treated control group. In the lung, no specific abnormal findings were evident, although focal vascular mineralization in the vascular wall of arteries was often found in a few rats with no difference between the control and the mixture treatment groups in the incidence and severity (**Figure 11**). In the nasal cavity, squamous metaplasia of the respiratory epithelium of the section level I was evident in one rat of the medium-dose group and all rats of the high-dose group, while the lesion was not observed in the non-treated control, vehicle, and the low-dose group. The lesion was characterized by replacement of respiratory epithelium by squamous epithelium with complete loss of cilia. This observation in the nasal cavity of mixture exposure group is clearly different from results of the SM single chemical inhalation study because any specific effects were not observed in SM only treated experiment.

**Table 2:** The summary of the histopathological results of rats repeatedly inhaled with the mixture of SM and propylene glycol (PG).

Groups histopathology	Groups	Control	Vehicle	Low	Middle	High
		0	PG1%	PG1%+SM1%	PG1%+SM5%	PG1%+SM20%
	**No. examined**	**4**	**4**	**4**	**4**	**4**


**Liver**					
No specific lesion		1 (25.0)	1 (25.0)	1 (25.0)	2 (50.0)	0 (0.00)
Cell infiltration, mononuclear cells,		3 (75.0)	3 (75.0)	3 (75.0)	2 (50.0)	4 (100)
multifocal
Grades; minimal		3	3	2	2	4
*mild*		0	0	1	0	0

**Lung**					
No specific lesion		3 (75.0)	2 (50.0)	3 (75.0)	3 (75.0)	3 (75.0)
Vascular mineralization, focal		1 (25.0)	1 (25.0)	1 (25.0)	0 (0.00)	1 (25.0)
Grades; minimal		1	1	1	0	1

**Nasal cavity**					
No specific lesion		4 (100)	4 (100)	4 (100)	3 (75.0)	0 (0.00)
Metaplasia, squamous cells, Line I		0 (0.00)	0 (0.00)	0 (0.00)	1 (25.0)	4 (100)
Grades; minimal		0	0	0	1	0
*mild*		0	0	0	0	4

**FIGURE 12:**
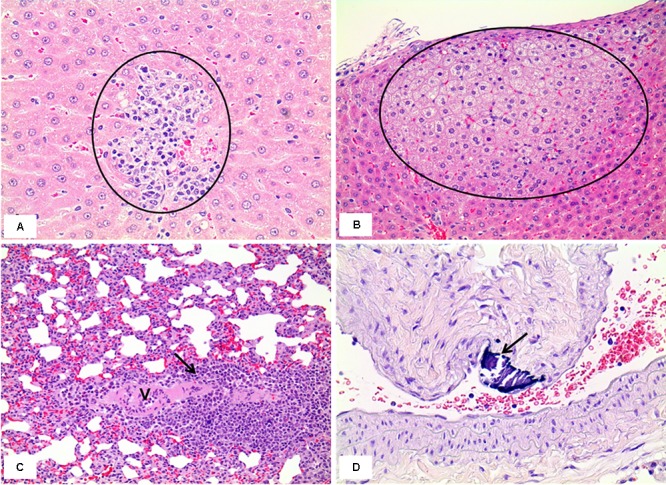
Histopathology of livers and lungs in the male rats exposed by inhalation of the mixture of SM and PG for 2 weeks in the recovery study. The results represent focal mononuclear cell infiltration (circle in **A**, the control group) and tension lipidosis (circle in **B**, the medium group) in the liver and perivascular mononuclear cell infiltration (arrow in **C**, the vehicle control group) and vascular mineralization (arrow in **D**, the low group) in the lungs. H&E staining. Magnification = ×400 for **A**, ×200 for **B**, **C**, and **D**.

**FIGURE 13:**
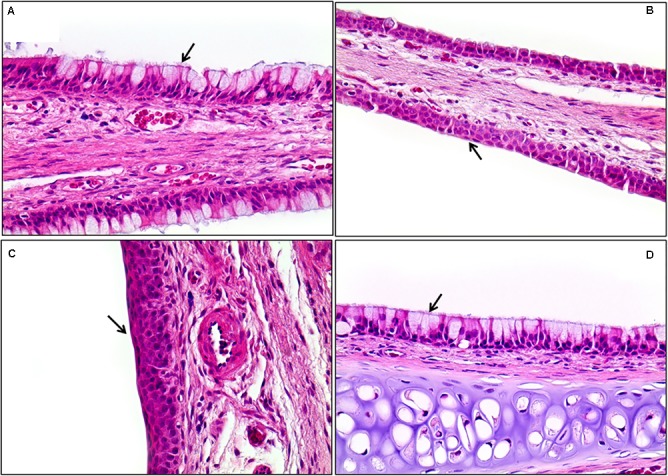
Histopathological staining of the nasal cavity in the male rats exposed to the mixture of SM and PG for 2 weeks by aerosol inhalation. Panels **(A–C)** are the results of exposure groups each defining for control, medium-, and high-dosage treatment, respectively. Panel **(D)** shows the recovery of high-exposure groups. Note the metaplasia of the respiratory epithelium into squamous cells in **(B)** and **(C)** (arrows). Compare these with normal respiratory epithelium composed of ciliated pseudostratified columnar epithelium with goblet cells [arrows in **(A)** and **(D)**]. Squamous cell metaplasia occurred in the front of the nasal cavity, but was completely recovered 7 days after exposure was stopped **(D)**. H&E staining. Magnification = ×400 for all images.

In the 1 week recovery study, various background lesions were found in a few rat livers of the control and the mixture treatment groups, including multifocal mononuclear cell infiltration, focal bile ductule hyperplasia, and tension lipidosis (**Figures 12A,B**). Similarly, vascular mineralization in the arterial wall, focal foamy macrophage aggregation, and perivascular mononuclear cell infiltration was observed in the lungs in the control and the mixture treatment groups without toxicological significance (**Figures 12C,D**). The squamous metaplasia, which occurred in the section level I of nasal cavity when exposed to the mixture at the medium and high dose for 2 weeks, was not evident 1 week after termination of the exposure (**Figure 13**).

## Discussion

Sodium metabisulfite is a widely used biocide, commonly as a preservative in food processing and consumer products (Zhang et al., 2015). PG is commonly used as a solvent in many chemical products (Pillai et al., 2014). Although various products used in our daily life are composed of chemical mixtures, only a few toxicity studies of chemical mixtures are conducted. Our study was performed to evaluate the toxicological effects of SM, PG, and their mixture both *in vitro* and *in vivo*.

Many studies have reported that the toxicological interactions, such as potentiation or antagonism may occur when two chemicals are mixed together (Lu et al., 2015; Nachtergael et al., 2015; Puckowski et al., 2017). In the present cell viability study, the administration of PG alone did not induce any cytotoxicity, but potentiated the toxicity of SM when administered as a mixture (at 200 μg/ml SM), evidenced by the significant decrease in cell viability compared with the treatment of SM only (**Figure 1**). A previous study has documented the potentiation of didecyldimethylammonium chloride (DDAC) toxicity by the mixture with ethylene glycol (EG) in BEAS-2B cells (Kim et al., 2015). Clonogenic assay is widely used to assess cell proliferation, and cells were exposed to test material for about 7 days (Masuda and Asahara, 2013; Martignani et al., 2015). In the present study, only 50 μg/ml SM resulted in 47% decrease in colony formation compared with the control group, which suggested that the chronic effects of SM were comparably higher than the acute exposure to A549 cells at the same dose (**Figure 2A**). In contrast to the MTT assay, however, we did not find a potentiation effect of SM by the addition of PG in the clonogenic assay (**Figure 2B**). Stepić et al. (2013) insisted that the potentiation character of chemical was a specific phenomenon, rather than a general effect, and that the reason was related to the chemical structures of the mixture. Although we found the potentiation of SM in the cell viability assay, only A549 cells were used, and only PG was mixed with SM in our study. Therefore, future studies should include follow-up works designed to evaluate the potentiation of SM in various kinds of cells when mixed with other chemicals.

Both PMNs and macrophages have an important role in the first-line defense against xenobiotics, through phagocytosis and the recruitment of cytokines (Djeu et al., 1986; Di Carlo et al., 2001; Wesselkamper et al., 2001). In the present study, we have revealed that the significant increase of PMNs in the high-dose group and medium-, high-recovery groups after exposure of SM and PG mixture. In both medium- and high-dose groups, the percent of PMNs was higher in the recovery groups than in the exposure groups, indicating the mixture of SM and PG could have persistent toxic effect in the lung. In addition, whereas there was no symptom in the rats of the SM single exposure, clinical symptoms such as nasal sounds, sneezes, and eye irritations were observed in the rats of the high SM+PG mixture exposure group. According to the histopathological results, SM alone was found not to give any toxic effects to the liver, lung, and nasal cavity; however, the mixture of SM and PG induced squamous metaplasia of the respiratory epithelium in the section level I of the nasal cavity, indicating the potentiating effect of PG in the SM toxicity in nasal cavity. The lesion, characterized by replacement of respiratory epithelium by squamous epithelium, is considered to be an adaptive change following repeated or prolonged insult. However, the lesion was completely recovered by 1 week discontinuation of the exposure to the mixture of SM and PG, indicating it was a reversible change depending on the exposure. The differences in toxicity between SM alone and SM+PG mixture could be explained by the distribution of aerosol particle, as the count of large-sized aerosol particles increased when the concentration of SM+PG increased. In detail, the increased number of particles with the size of 1 μm and over was predominantly observed in the SM+PG exposed chamber compared to the SM single chemical exposed chamber, suggesting that aggregation of chemicals possibly be occurred in the generated aerosols. As the size increases, the particles could deposit easily in the nasal cavity of rats, resulting in such histopathological alterations in the high-dose mixture groups compared to the SM single exposed rats (Shang et al., 2015). Other lesions observed in the liver, lung, and nasal cavity in the termination and recovery studies were not related to the exposure of SM and PG mixture, because they occurred not only in the treatment groups but also in the non-treated or vehicle control groups without significant difference in their incidence and severity.

People are generally exposed to the mixture of chemicals rather than single chemical in their daily lives. However, we could not easily assume the toxicity of mixture because chemical interactions could occur through various ways (Deneer, 2000; Jarvis et al., 2014). Many researchers have insisted that the effects caused by a single chemical may differ when they are mixed with other chemicals (Fortoul et al., 2005; Kortenkamp, 2014), also evidenced in our present study. Our study revealed a difference in the toxic effects between the single chemical and the mixture of chemicals, although it has some limitations owing to the few endpoints of cytotoxicity and the short exposure periods of inhalation. Future studies should therefore include follow-up of various *in vitro* tests and longer-term inhalation studies.

In summary, it was found in the present study that PG potentiated the toxicity of SM in human lung epithelial cells, also observed in the 2-week inhalation study in rats. Therefore, the present study clearly showed the potentiation of SM toxicity through addition of PG both in *in vitro* and *in vivo* systems.

## Author Contributions

PK, SY, and H-MK conceived and designed this study. JY, B-IY, and IS wrote the manuscript. JY, IS, Y-ML, HK, E-JK, and BL did experiments of cytotoxicity and inhalation toxicity. B-IY performed the experiments of histopathology in rats. D-HL measured the concentrations of SM and PG.

## Conflict of Interest Statement

The authors declare that the research was conducted in the absence of any commercial or financial relationships that could be construed as a potential conflict of interest. The reviewer SK and handling Editor declared their shared affiliation.

## Footnotes

1 http://householdproducts.nlm.nih.gov
